# DREAM: an adaptive, randomised, placebo-controlled trial of duloxetine for reducing leg pain in people with chronic sciatica—trial protocol

**DOI:** 10.1136/bmjopen-2024-096796

**Published:** 2024-12-31

**Authors:** Hanan McLachlan, Christopher G Maher, Chung-Wei Christine Lin, Laurent Billot, Richard O Day, Rowena Ivers, Martin Underwood, Andrew J McLachlan, Bethan Richards, Nanna B Finnerup, Giovanni E Ferreira, DREAM Study Group, Giovanni E Ferreira

**Affiliations:** 1 Institute for Musculoskeletal Health, Sydney Local Health District, Sydney, New South Wales, Australia; 2 Sydney School of Public Health, Faculty of Medicine and Health, The University of Sydney, Sydney, New South Wales, Australia; 3 The George Institute for Global Health, Faculty of Medicine and Health, University of New South Wales, Sydney, New South Wales, Australia; 4 Department of Clinical Pharmacology and Toxicology, St Vincent's Hospital Sydney, Sydney, New South Wales, Australia; 5 St Vincent's Clinical School, Faculty of Medicine, University of New South Wales, Sydney, New South Wales, Australia; 6 Graduate School of Medicine, Faculty of Science, Medicine and Health, University of Wollongong, Wollongong, New South Wales, Australia; 7 Warwick Clinical Trials Unit, Warwick Medical School, University of Warwick, Coventry, UK; 8 University Hospitals Coventry and Warwickshire NHS Trust, Coventry, UK; 9 Sydney Pharmacy School, Faculty of Medicine and Health, The University of Sydney, Sydney, New South Wales, Australia; 10 Rheumatology Department, Institute of Rheumatology and Orthopaedics, Royal Prince Alfred Hospital, Camperdown, New South Wales, Australia; 11 Danish Pain Research Centre, Department of Clinical Medicine, Aarhus University, Aarhus, Denmark

**Keywords:** Neurological pain, Chronic Pain, Clinical trials, Musculoskeletal disorders

## Abstract

**Introduction:**

Sciatica is a debilitating condition that often becomes chronic, and for which there are few effective treatment options. Treatments such as the anti-depressant duloxetine have shown promise, but the evidence is inconclusive. We are describing a high quality, definitive trial to investigate the efficacy, safety and cost-effectiveness of duloxetine in chronic sciatica.

**Methods and analysis:**

The duloxetine for chronic sciatica (DREAM) trial is a randomised, superiority, parallel-group, placebo-controlled, triple-blinded (participant, clinician, assessor) trial with an adaptive group sequential design investigating the efficacy and safety of duloxetine in participants with chronic sciatica of at least 3 months duration. Participants will be randomised at a 1:1 ratio to duloxetine or placebo. 332 participants will be recruited on presentation to general practices, specialist clinics and hospital emergency departments or from hospital in-patient wards and from the community. In the active treatment group, participants will receive duloxetine 60 mg per day for 12 weeks, including 1 week of titration at 30 mg/day. The treatment phase will be followed by a 2-week tapering phase where they will receive duloxetine 30 mg/day. Participants will be followed-up for 1 year, with outcomes being measured 4, 8, 12, 16, 26, and 52 weeks post-randomisation. The primary outcome is leg pain intensity at 12 weeks post-randomisation. Secondary outcomes include back pain intensity, disability, time to recovery, quality of life, depressive and anxiety symptoms, and sleep disturbance. Adverse events will be recorded, and a cost-effectiveness analysis will be conducted.

**Ethics and dissemination:**

Ethical approval has been granted by the University of Sydney Human Research Ethics Committee. Trial results will be disseminated by publications, conference presentations and via the media.

**Trial registration number:**

ACTRN12624000919516.

**STRENGTHS AND LIMITATIONS OF THIS STUDY**

- DREAM is a two arm, triple-blinded (participant, clinician and assessor) randomised controlled trial of duloxetine compared with placebo for reducing leg pain in patients with chronic sciatica.
- DREAM uses an adaptive group sequential design with two interim analyses.
- Despite blinding of the study medication, there is a risk that participants may suspect they were randomised to the active treatment arm given the known adverse effects of duloxetine (eg, nausea, dry mouth).

## Introduction

Sciatica is the common term used to describe painful lumbosacral radiculopathy. It is clinically defined as pain that radiates down the leg in a dermatomal distribution and is accompanied by neurological deficits (eg, sensory loss, weakness or reflex loss) in the corresponding dermatomal or myotomal distribution.^1 2^ Sciatica is typically considered to have a favourable prognosis, but this assumption is not supported by evidence.^3^ Half of those who develop sciatica do not improve after 1 year; and, for nearly 40% of these, their persistent pain is of at least moderate intensity.^4^ The burden of sciatica is also disproportionally high compared with those with back pain only. People with sciatica have more pain and disability, poorer quality of life and use more healthcare resources than those with back pain only.^5^

Pharmacological treatments are a common component of the clinical management of sciatica.^6 7^ However, the most used pharmacological treatments for sciatica are either known to be ineffective or their effectiveness is unclear. For example, while opioid analgesics are the most commonly used pain relieving medicine for sciatica, their efficacy for sciatica is uncertain and opioid-related harms may outweigh their benefits.^8^ Non-steroidal anti-inflammatory drugs have small effects on pain compared with placebo for acute sciatica,^9^ but there are no trials in chronic sciatica,^10^ and their use increases the risk of vascular and gastrointestinal events.^11^ Anti-convulsants (eg, pregabalin) are commonly used, but these medicines are reported to be ineffective for managing sciatica.^12 13^

Anti-depressants are commonly used to treat chronic sciatica, but the evidence is inconclusive.^14 15^ In a 2021 systematic review, we found evidence suggesting that some anti-depressants, such as those from the serotonin-noradrenaline reuptake inhibitor (SNRI) class, may be effective for chronic sciatica and provide clinically worthwhile reductions in pain.^15^ However, estimates were based on three small (n=96), low-quality trials, and the certainty of evidence ranged from low to very low. Of the SNRI anti-depressants investigated for the management of sciatica, duloxetine appears to be the most promising. Duloxetine has been shown to be effective for the largest number of conditions among all other anti-depressants^14^ and is better tolerated than other anti-depressants commonly used for chronic pain conditions.^16^,^19^ Unlike opioids, duloxetine use has not been associated with the risk of misuse and addiction.^20^

Duloxetine is a potent inhibitor of serotonin and norepinephrine uptake, two key neurotransmitters involved in pain modulation.^21 22^ The analgesic effect of duloxetine has been attributed to both central and peripheral mechanisms based on research in animal models.^18^ These studies suggest a central mechanism that is rapid and requires descending noradrenergic inhibitory controls from the brain to the spinal cord plus a peripheral mechanism that is delayed and relies on the anti-neurogenic inflammation properties of duloxetine.^18^ The ability of duloxetine to reduce neurogenic inflammation makes it a promising pharmacological treatment for sciatica, a condition characterised by neuropathic pain.^23^ Duloxetine is also a low-cost treatment. For example, in 2024, the out-of-pocket cost in Australia for a 12 week treatment regimen of 60 mg per day followed by 2 weeks of tapering at a dose of 30 mg per day is estimated to be as low as $A91 (calculated based on dispensed price of 3×28 APO-Duloxetine 60 mg enteric capsules and 1×28 APO-Duloxetine 30 mg enteric capsules).^24 25^ Although not currently approved by the Therapeutic Goods Administration in Australia for the treatment of back pain, anti-depressants are commonly prescribed ‘off-label’ for back pain.^26^

To resolve the current uncertainty, we plan to conduct the duloxetine for chronic sciatica (DREAM) trial, a randomised placebo-controlled trial to investigate the efficacy and safety of duloxetine for chronic sciatica. The DREAM trial will provide high-quality evidence on the efficacy of duloxetine for sciatica, addressing a key research priority in the field.^27^

The primary aim of DREAM is to investigate the efficacy of duloxetine, compared with placebo, on leg pain intensity in people with chronic sciatica. The secondary aims are to investigate its efficacy in improving other key patient reported outcomes (including low back pain, disability, quality of life, symptoms of depression, anxiety and sleep disturbance) and its safety and cost-effectiveness in this indication.

## Methods and analysis

### Design

DREAM is a randomised, superiority, parallel-group, placebo-controlled, triple-blinded (participant, clinician, assessor) trial with an adaptive group sequential design. DREAM was designed to investigate whether taking 60 mg of duloxetine daily for 12 weeks can reduce leg pain intensity in individuals with chronic sciatica. The trial has been prospectively registered on the Australia and New Zealand Clinical Trials Registry (ACTRN12624000919516). This paper details the trial protocol and follows the Standard Protocol Items: Recommendations for Interventional Trials 2013 Statement recommendations.^28^

### Study setting and identification of participants

**Figure 1:**
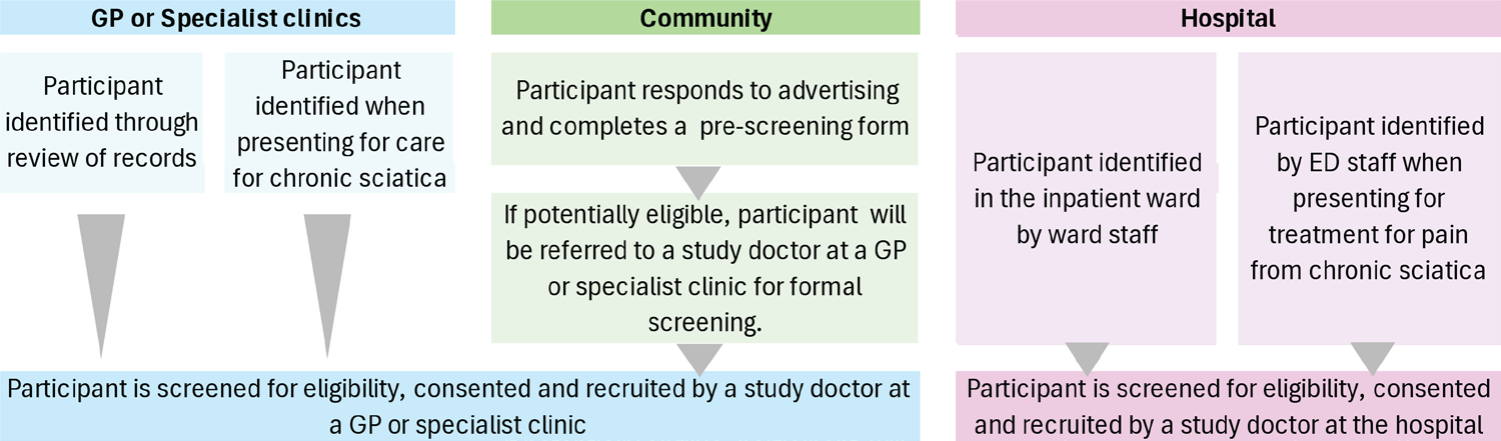
Recruitment pathways ED, emergency department; GP, general practitioner.

The DREAM trial will be conducted across various healthcare settings, including general practices, specialist outpatient facilities (such as rheumatology clinics), hospital emergency departments and inpatient wards. Registered medical practitioners will be invited to participate as study doctors. Participants will be screened and, if eligible, recruited to the study when they are seen by a participating study doctor. Potential participants will also be identified through community advertising (eg, on social media). These participants will be referred to a study doctor to be screened and, if eligible, recruited to the study. Regardless of the clinical setting, all study sites will follow the same study procedures. Consultations with the study doctor may be face-to-face or via telehealth. The potential recruitment pathways are shown in figure 1. Participant recruitment is expected to run between December 2024 and June 2027 with the final follow-up assessment in June 2028.

### Eligibility criteria

Adults living with chronic sciatica will be screened for eligibility by a study doctor. A screening form will be completed for each potential participant to confirm eligibility for the study. Eligibility criteria are described in box 1.

Box 1 **Eligibility criteria**

**Inclusion criteria**

- Adults (≥18 years) with radiating pain into one leg in a dermatomal distribution.
- Leg pain duration of at least 3 months.
- Evidence of nerve root involvement, defined by the presence of at least one of the following clinical signs in the corresponding distribution: myotomal weakness and/or diminished reflex and/or sensory deficit and/or imaging evidence of nerve root impingement that is consistent with the clinical presentation.
- Leg pain that is at least moderate in intensity at the time of enrolment by a study doctor (as measured by a modified version of item 21 in the 36-Item Short Form Survey Instrument).
- An adequate understanding of English or the availability of interpretation services for the participant to complete the trial.

**Exclusion criteria**

- Known or suspected malignancy or specific pathologies in the spine (eg, fracture, cauda equina syndrome).
- Having had spinal surgery or an interventional procedure (eg, epidural injection) in the preceding 6 months.
- Scheduled to have a spinal procedure (eg, spinal surgery, epidural injection) within 12 weeks at the time of enrolment.
- Currently taking (or taken in the last 2 weeks) any anti-depressant for any condition.
- Any contraindications to duloxetine: known hypersensitivity to duloxetine, known acute or chronic liver disease, concomitant use with CYP1A2 inhibitors (eg, fluvoxamine) or concomitant use of monoamine oxidase inhibitors (eg, moclobemide), or use that has ceased less than 2 weeks ago.
- Known history of chronic kidney disease stage 4 or above.
- Precautions for use of duloxetine as specified by the Product Information where risks outweigh potential benefits (eg, depressive symptoms for which treatment is required as judged by the study doctor, bipolar disorder, history of seizure disorder, etc).
- Previous severe adverse reaction to duloxetine (eg, serotonin syndrome) as judged by the study doctor.
- For females: pregnancy, breastfeeding or planning conception during the treatment period.

### Participant timeline

**Figure 2:**
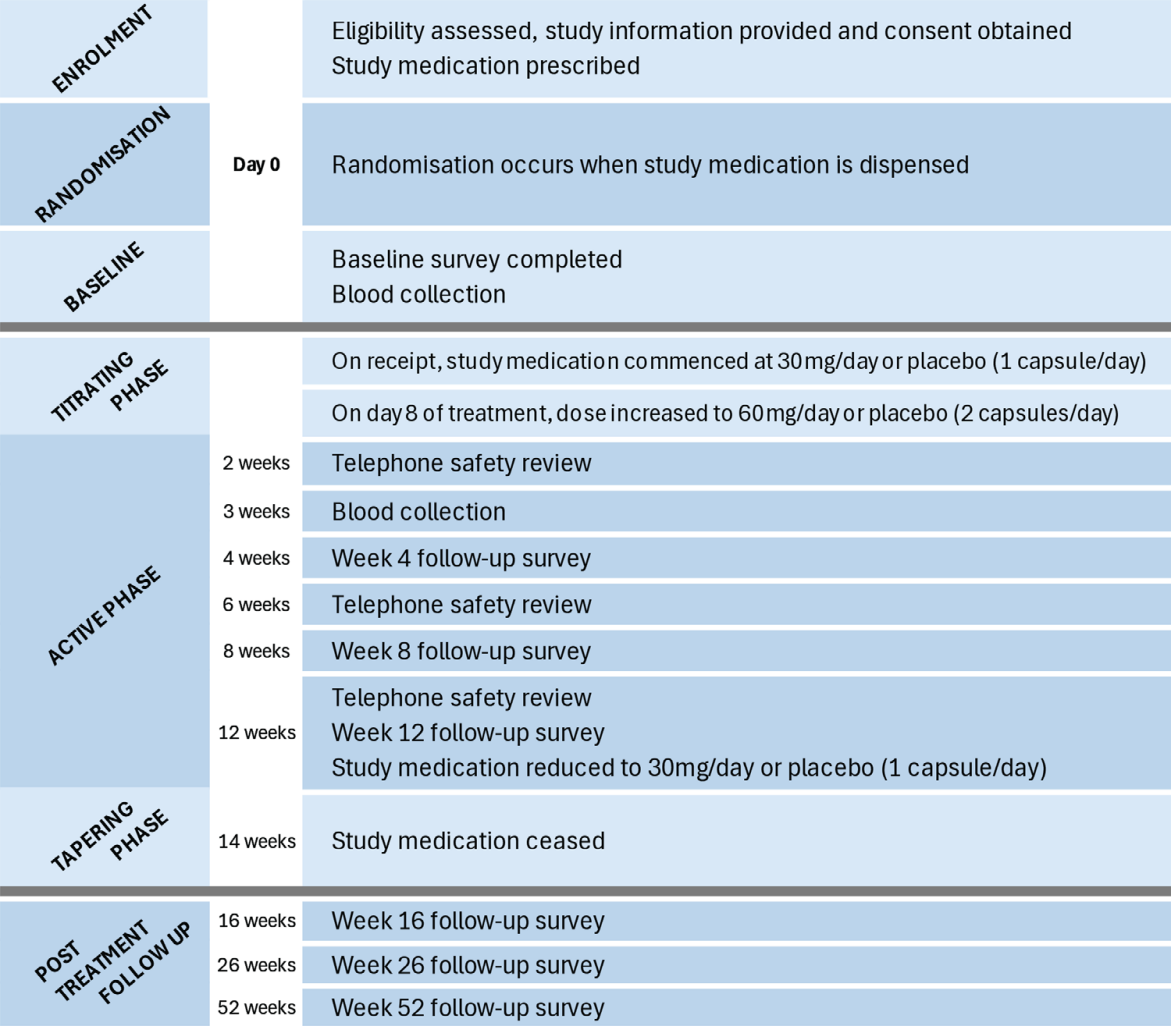
Participant timeline

Participants will have an initial consultation with their study doctor and up to two follow-up consultations, if required. These follow-up consultations can happen anytime from week one post-randomisation to week 14 post-randomisation. The participant timeline is shown in figure 2.

### Assignment of interventions: allocation/randomisation

An independent statistician not involved in any other aspect of the study will generate the randomisation sequence a priori using a computerised random number generator. Random allocation to either duloxetine or placebo will occur in a 1:1 ratio using randomly permuted blocks of various sizes. Study medication packs will be manufactured and labelled according to the randomisation sequence by the investigational medicinal product management company, Syntro Health. Medication packs will be allocated to the various recruitment settings (ie, general practice or specialist clinics, hospital or community) in complete block sizes. The study medication packs will be dispensed direct to the participant via a central pharmacy service provided by Syntro Health, except for those participants recruited from the hospital setting whose study medication will be dispensed via the hospital’s clinical trials pharmacy. The study medication packs will be dispensed sequentially from within the blocks allocated to the respective recruitment setting.

The participant will be enrolled into the study after the informed consent process has been completed and the participant has met all inclusion criteria, and none of the exclusion criteria and study medication has been prescribed for them. The participant will be dispensed a study medication pack with a unique study enrolment number. Randomisation will occur when the medication pack is allocated to the participant during dispensing.

### Blinding

Allocation to treatment group will be concealed, and the active and placebo medicines will be identical in appearance and taste, ensuring blinding of the participant, study doctor, assessor and all study personnel including the research team and the steering committee. We will measure the success of blinding by asking participants to guess their group allocation at the end of the treatment period.

To maintain the overall quality and rigour of the study design, unblinding should only occur in exceptional circumstances when knowledge of the actual treatment is essential for further clinical management of the participant, such as with Serious Adverse Events. The decision to unblind a participant will be made in consultation with the clinicians involved in the participant’s management (including the study doctor), the independent Medical Monitor, and the Steering Committee.

If unblinding is deemed to be necessary, the study team will facilitate contact between the clinicians involved in the participant’s management and the independent statistician who generated the randomisation sequence, and care will be taken to ensure that only the study personnel who will be involved in further management of that participant will be unblinded. Unblinding will not necessarily be a reason for the participant to discontinue the study.

After the final participant has completed the study, the study doctors, research team and Steering Committee will remain blinded until the data analysis and interpretation is complete.

### Intervention

Participants in both groups will receive guideline-recommended advice from their study doctor, consisting of information on the nature of sciatica, its prognosis, encouragement to continue with normal activities, importance of avoiding bed rest and to avoid opioid medicines.^27 29^ To ensure consistency in how advice is delivered, study doctors will be trained in the study protocol and receive ongoing support from research team throughout the trial.

#### Study medication

Study participants will be provided with the blinded study medication pack containing either the active study medication (duloxetine 30 mg enteric-coated capsules) or matching placebo capsules manufactured and labelled to Good Manufacturing Practice standard by Syntro Health (Victoria, Australia).

Participants randomised to the active arm of the study will receive a 12-week course of oral duloxetine followed by a 2-week tapering phase. The starting dose will be duloxetine 30 mg/day for 1 week (one 30 mg capsule per day), increasing to 60 mg/day for 11 weeks (maintenance phase: two 30 mg capsules per day). In the 2-week tapering phase, they will receive 30 mg/day (one 30 mg capsule per day) for 2 weeks before treatment is discontinued.

The duloxetine regimen of 60 mg one time per day was chosen as it is the recommended dose for chronic pain and diabetic peripheral neuropathic pain.^30^ There is no evidence, including evidence from our review,^15^ that higher duloxetine doses (eg, 120 mg/day) confer additional benefits, but they do increase the incidence of adverse events and treatment discontinuation.^30^ The 12-week duration of the treatment is consistent with that reported in trials of duloxetine for other pain conditions.^14^ A 2-week tapering period is consistent with recommendations for the Australian population to minimise the risk of withdrawal symptoms.^20^

Participants randomised to the placebo arm of the study will receive the equivalent number of matching placebo capsules throughout the 14 weeks of the treatment phase.

Participants will be contacted by telephone at weeks 2, 6 and 12 by a blinded research team member to monitor treatment and collect adverse events data. This researcher, who will have nursing or pharmacy qualifications, may recommend participants to visit their study doctor for dose modification if they are experiencing troublesome adverse events or there are concerns regarding the participant’s safety. The responsible researchers will be trained and provided guidance regarding events and concerns that should receive medical attention. Study doctors, who also remain blinded, may make modifications to the treatment regimen if required. Changes and a justification for the changes will be noted in a treatment monitoring form. Participants will be allowed two follow-up visits to their study doctor during the active treatment phase, if required.

Participants experiencing adverse effects will be allowed to reduce the dose of study medication in the treatment phase to one capsule per day (30 mg/day in the duloxetine arm) if considered appropriate by the study doctor. Likewise, if confirmed by their study doctor, participants will be allowed to start the tapering phase earlier if they recover before the 12-week period is completed. Recovery is defined as seven consecutive days with leg pain intensity ≤1 out of 10. Duloxetine dose escalation above 60 mg per day is not permitted in this trial.

Once a participant has given informed consent and has been recruited to the trial, a prescription will be sent electronically to the central pharmacy at Syntro Health. The central pharmacy will dispense and dispatch the study medication packs direct to the participant’s preferred address. Medication packs will be dispensed in sequential order. Randomisation occurs when the study medication pack is assigned to a participant during dispensing. The medication pack will include four bottles of blinded study medication: three bottles each containing 54 capsules and one bottle containing 14 capsules, plus a copy of the consumer medicines information leaflet for duloxetine. The smaller bottle will be designated for the 2-week tapering period. The medication pack and individual bottles will be labelled in accordance with labelling requirements for investigational medicines in Australia. In addition, the labels will include instructions on when to increase and decrease the number of capsules taken per day.

To monitor compliance with the study medication regimen, study doctors will be asked to confirm the study medication prescribed, and participants will be asked to record their intake in a daily diary. On cessation of treatment either at 14 weeks or earlier, if appropriate, participants will be asked to return any unused medicines to the research team for counting using a prepaid satchel.

#### Concomitant treatments

All participants may receive additional care during the treatment period as deemed appropriate by their study doctor. This may include physical or manual therapies or other medications except other anti-depressants or medications that should not be used in combination with duloxetine. Study doctors will be recommended to refrain from scheduling interventional procedures during the 14-week treatment period.

Participants will be advised that they should not commence any prescription medicine while they are taking the study medication without discussion with the study doctor. This provision does not apply if urgent pharmacotherapy is required in a medical emergency.

To minimise the risk of inappropriate concomitant treatments (eg, medicines that also have a serotonergic effect and that could increase the risk of serotonin syndrome. eg, tramadol, or potent inhibitors of CYP1A2, eg, ciprofloxacin), participants will be issued a Study ID card which they should present to their treating healthcare practitioners. The card will indicate the participant is receiving blinded study medication which may be duloxetine. The card will include the research team’s contact details.

We will record the use of all concomitant medications for sciatica, including analgesic medications, at each follow-up survey. Participants will be allowed to use concomitant analgesia provided the medication is not contraindicated with duloxetine.

### Outcomes

The outcomes for this study were informed by a core set of outcomes recommended for back pain trials and consultation with consumers.^31 32^ Outcome measures, unless otherwise stated, will be collected by questionnaire at baseline, 4, 8, 12, 16, 26 and 52 weeks.

#### Primary outcome

The primary outcome is leg pain intensity (average intensity over the past 24 hours) measured on a 0–10 numerical rating scale (NRS). The primary timepoint is at week 12, when the participant will have completed 12 weeks of treatment and is about to commence tapering of the study medication.

#### Secondary outcomes

The key secondary outcome is disability measured by the Roland Morris Disability Scale for Sciatica.^33^

The following will also be recorded as secondary outcomes:

- Low back pain intensity (average intensity over the past 24 hours) measured on a 0–10 NRS.
- Time to recovery measured by a pain diary. Participants will be asked to record the average daily leg pain score in a pain diary for the duration of the treatment period using the 0–10 NRS. Recovery is defined as seven consecutive days with leg pain intensity ≤1 out of 10. Data will be censored at 14 weeks or if participants meet the definition of recovery, whichever occurs first. If recovery has not occurred by 14 weeks, participants will be asked at subsequent follow-up assessments (26 and 52 weeks) whether they have recovered or not and the recovery date (if available).
- Health-related quality of life measured by the EQ-5D-5L, an instrument developed by EuroQol which assesses 5 dimensions of health with 5 levels of severity for each dimension.^34^
- Depressive symptoms measured by the Patient Health Questionnaire-9.^35^,^37^ This will be collected at baseline and week 8.
- Anxiety symptoms measured by the Generalised Anxiety Disorder 7-item.^38^ This will be collected at baseline and week 8.
- Sleep disturbance measured by the 8-item Patient-Reported Outcomes Information System Sleep Disturbance Short Form.^39^ This will be collected at baseline and week 8.
- Global perceived effect (GPE) scale. GPE is measured on a −5 (vastly worse) to +5 range (completely recovered).^40^

The following outcomes will also be recorded:

- Adverse events (defined under the Harms section) will be collected by self-report at weeks 4, 8, 12 and 16 via the online or phone follow-up questionnaires. Additionally, the research team will contact participants by phone at weeks 2, 6 and 12 to assess whether they have experienced any adverse events in general and in particular whether they have experienced any of those on the 21-item Antidepressant Side-Effect Checklist.^41^
- Work absenteeism will be measured in a questionnaire collected at all time-points. Participants will be asked to estimate the number of days off paid work in the period since the previous follow-up survey with the following question: ‘In the last 4 weeks (*or period since the last study follow-up*), did you miss any days off your normal paid work due to your sciatica?’.^42^
- Adherence to study medication will be measured by the participants’ self-report of daily medication intake, as recorded in an online or paper diary, and by comparing the count of returned medications against the doctor’s prescription.
- Healthcare use, including healthcare consultations, diagnostic and therapeutic procedures, imaging and pathology services, and prescription medicines, will be collected using administratively linked data from the Australian Medicare Benefits Schedule (MBS) and Pharmaceutical Benefits Scheme (PBS), respectively, and each follow-up questionnaire.

### Exploratory investigations

Where possible, we will collect blood samples at baseline and week 3 to measure the following:

- Duloxetine serum trough concentration at week 3.^43 44^
- Pharmacogenomic testing (eg, CYP2D6) at baseline.^45^,^47^
- High-sensitivity c-reactive protein at baseline and 3 weeks.^48^

### Sample size

A sample size of 266 patients (133 per group) provides 90% power to detect a 1-point difference in leg pain intensity, measured on a 0–10 scale, between the duloxetine and placebo groups. The calculation assumes a SD of 2.5 based on previous duloxetine trials for back pain^15^ and uses a two-tailed alpha level of 5%. This sample size also provides more than 90% power to detect a clinically worthwhile difference of 3 points on the Roland-Morris Disability Sciatica Questionnaire, which is the trial’s key secondary outcome, assuming a conservative SD of 6.

**Figure 3:**
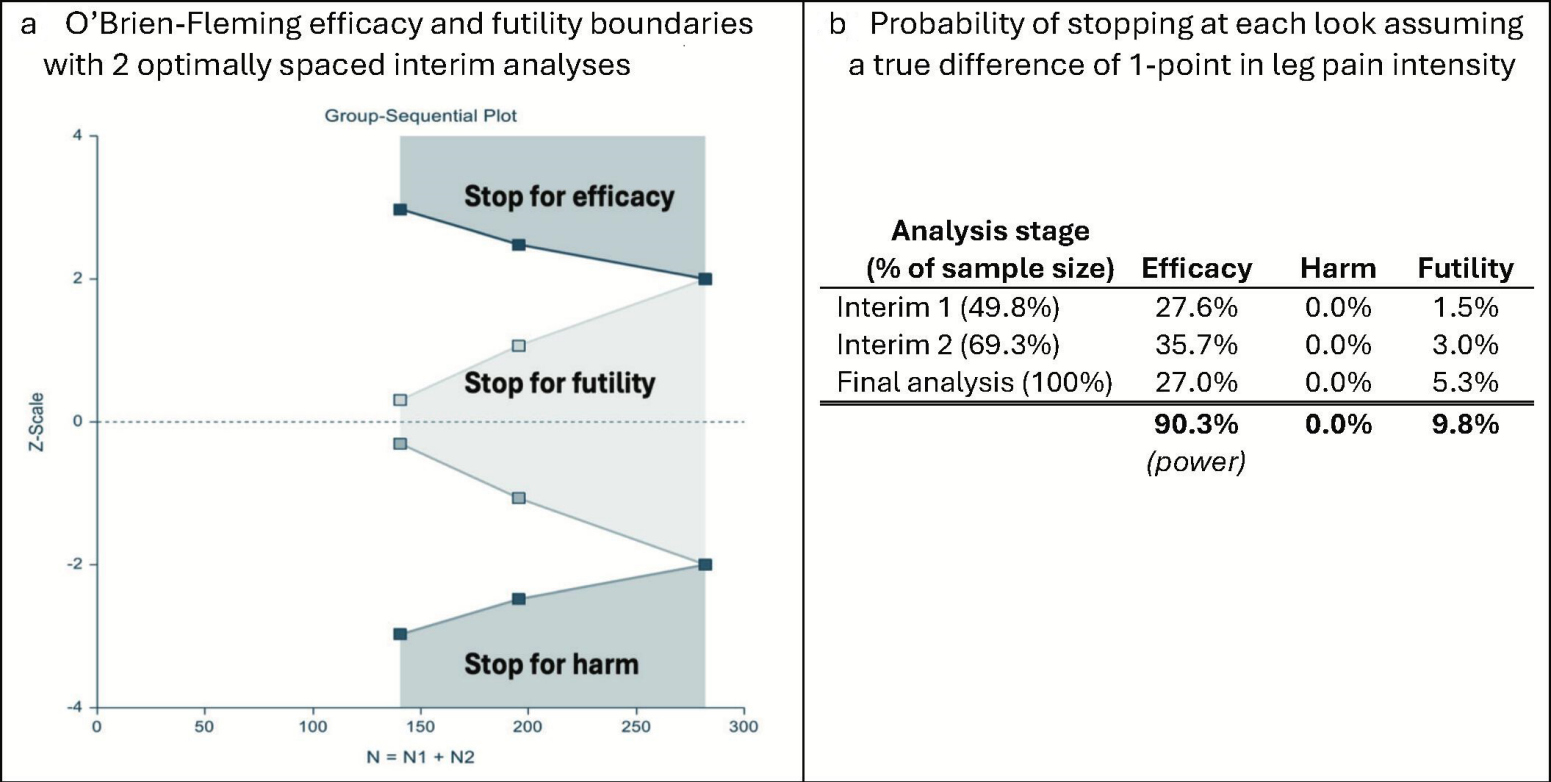
Stopping boundaries (**a**) and probability of stopping at each interim analysis under the alternative hypothesis (**b**)

To increase the chance of finding the correct answer early and minimise the expected sample size, we will use an adaptive group sequential design with O’Brien-Fleming efficacy and non-binding futility stopping boundaries.^49^ This approach is based on flexible ‘spending functions’ which adjust the level of alpha and beta spent based on the number of looks. It has the advantage that the exact number and timing of interim analyses does not need to be prespecified. Based on the 10 000 trial simulations with up to three optimally spaced looks (two interim analyses), this approach leads to an expected total sample size of 208 participants (an expected saving of 58 participants, or 22% of the sample size compared with a non-adaptive design) and, in the case of no early stopping, a maximum sample size of 282 participants (only 16 (6%) more participants than with a non-adaptive design) (figure 3). Allowing for a conservative estimate of 15% of dropouts based on tolerability data from our previous work, we will aim to recruit a maximum sample size of 332 participants.

### Strategies for achieving adequate participant enrolment

We plan to recruit up to 100 study sites to screen and recruit participants. We will run advertising campaigns on social media platforms where respondents will be prescreened by the research team who will refer suitable respondents to the study sites. Other strategies to ensure the recruitment target is achieved will include regular phone, email and visit contact of the study sites by the research team to monitor progress and provide support, provision of study posters and brochures for the study sites to display, streamlined study procedures (screening, recruitment and follow-up) to reduce the workload of clinicians and participants, and reimbursement of study sites for the time spent on trial-related tasks.

### Data collection methods

A custom-built study database will be created in REDCap. Following an established process,^13 50^ we will use electronic data capture where data collected by phone will be directly entered into the database by research assistants, and data completed by participants online (via a secure website) will be automatically transcribed into the database.

### Data management

The integrity of data will be closely monitored for omissions and errors in the secure REDCap study database hosted by the University of Sydney.

### Statistical methods

Data analysis will be conducted blinded on an intention-to-treat basis that is, by analysing patients according to their randomised group regardless of post-randomisation events. It will be guided by a prespecified statistical analysis plan which will be made public prior to database lock. Analyses will be conducted and validated using randomly scrambled group allocations before unblinding.

#### Primary analysis

The primary analysis will consist of a repeated-measures linear mixed model including leg pain scores at each post-randomisation follow-up survey, thus allowing every participant with at least one measurement to be included in the primary analysis. The model will include the randomised intervention, the baseline leg pain score and the follow-up period as a categorical variable. An interaction term between visit and intervention will be included to allow the intervention effect to be estimated separately at each time point. The primary endpoint will be the main effect of the intervention at 12 weeks post-randomisation and will be estimated from the model as the mean difference and its 95% CI. Multiple imputation will be performed to handle missing data if required.

#### Secondary analysis

The same model will be used to obtain estimates of the main effect of duloxetine at other time points for the primary outcome and for all time points for the secondary outcomes of a continuous nature. Adverse events will be classified, and the proportion of patients with adverse events and serious adverse events overall and within each category will be compared between groups using the Chi-square test.

#### Tertiary analysis

We will estimate the effect of the duloxetine regimen with full compliance using Complier Average Causal Effect analysis.^51^

#### Cost-effectiveness analysis

Two analyses will be conducted using the health system perspective: a cost-effectiveness analysis using the primary outcome as a measure of effectiveness and a cost-utility analysis where quality-adjusted life-years will be calculated using the EQ-5D-5L data collected at each follow-up. To obtain costs, administratively linked data will be used from the MBS for health services and the PBS for prescription medications. Participant questionnaires will identify the number of over-the-counter (OTC) medications and hospitalisations. OTC medications will be costed based on pharmacy websites and hospitalisations using the Independent Health and Aged Care Pricing Authority cost weights from the National Hospital Cost Data Collection. Bootstrapping will be used to calculate the confidence intervals around the incremental cost-effectiveness ratios. Sensitivity analyses will test uncertainty in key parameters such as the selection of cost weights.

### Data monitoring

An independent Data Safety Monitoring Board (DSMB) will be appointed by the Steering Committee. The primary objective of the DSMB is to review unblinded safety (adverse event and serious adverse events) data and advise the DREAM Steering Committee Chair if any change to the study is recommended. The DSMB will also review data from the pre-planned interim analyses and recommend whether the trial should be stopped early according to the pre-specified group sequential design.

### Harms

Adverse events (AEs) and serious AEs (SAEs), both defined below, will be assessed at weeks 4, 8, 12 and 16 by self-report in the follow-up questionnaires and at weeks 2, 6 and 12 via direct questioning using the 21-item Antidepressant Side-effect Checklist.

#### Adverse events

AEs are defined as any untoward medical occurrence in a participant administered the study medication. It does not necessarily have a causal relationship with the treatment.

#### Serious adverse events

SAEs will be defined as any untoward medical occurrence that results in death; is life threatening; requires hospitalisation or prolongation of the existing hospitalisation; results in persistent or significant disability or incapacity; is a congenital abnormality or birth defect; and is a medically significant or important event or reaction. SAEs will be reported to an independent medical monitor for assessment and to the ethics committee (and if they meet the criteria to the regulatory body) within the required timelines.

### Auditing

No formal auditing is planned. However, if required, independent auditing of core trial processes and documents will be arranged.

### Patient and public involvement (consumer engagement)

The development of the protocol was informed by consumer and clinician engagement. The Deputy CEO of Painaustralia (Monika Boogs) is an Associate Investigator on DREAM and ran a focus group with 15 consumers which informed decisions on the treatment regimen, outcome selection and collection. We consulted 54 clinicians (37 general practitioners, 12 rheumatologists, four pain specialists, and one spine surgeon) whose feedback informed decisions on the treatment regimen, population, and outcomes to be collected.

## Ethics and dissemination

### Research ethics approval

Ethics approval has been granted by the Human Research Ethics Committee, The University of Sydney (Project identifier 2024/HE000160).

### Protocol amendments

Any modifications to the protocol which may affect the trial design and conduct, or the potential benefits or harms to the participants, will require a formal amendment to the protocol. Such amendments will be agreed on by the Steering Committee and approved by the ethics committee prior to implementation.

### Consent

Study doctors will be trained on the informed consent process and will explain the study to potential participants who will also receive a patient information sheet and consent form (either in paper or electronic form). Study doctors, with assistance from the study team where necessary, will also answer any questions that are raised by potential participants and obtain written consent from those willing to participate in the study. At any stage, participants can withdraw consent without repercussion.

### Confidentiality

All study data will be stored securely in either locked file cabinets (paper files) or electronically (electronic database files) with access granted only to the study team. Where required, study doctors will have access to study data collected from the participants they are responsible for, only after consent from the participants.

### Access to data

The Steering Committee will have access to the final dataset, which may be provided to a statistician to assist with data analysis if required. To ensure confidentiality, the final dataset will contain deidentified information only.

### Ancillary and post-trial care

The cost of treatments outside the study treatment will not be borne by the study. Any post-trial care, including continuation or recommencement of an anti-depressant, will be determined by the participants and their clinician, whether they are study doctors or other qualified clinicians.

If non-negligent harm associated with the protocol occurs, participants will be covered by professional indemnity and clinical trials insurance of the trial. This will include cover for additional healthcare, compensation or damages.

### Dissemination policy

The main study results will be submitted for publication in a scientific journal, presented at relevant professional conferences and incorporated into evidence syntheses, guidelines and point of care recommendations. The results will also be disseminated to the media, public and policymakers. Authorship eligibility guidelines of publications arising from the study will align with those outlined by the International Committee of Medical Journal Editors (http://www.icmje.org/). There are no plans to use professional writers.

## Discussion

Each year thousands of Australians suffer with sciatica, and many of these have chronic pain. For those with chronic sciatica, there are very limited treatment options—none are simple, low cost and provide meaningful reductions in their levels of pain. We identified that duloxetine is a promising treatment for chronic sciatica, but there is an urgent need for a high-quality trial that will provide a definitive answer to this question.

This manuscript describes the design of the DREAM study which aims to address this gap in evidence. The DREAM study, a randomised, double-blind, placebo-controlled trial, will evaluate the effectiveness, safety and cost-effectiveness of duloxetine 60 mg per day in chronic sciatica when taken for 12 weeks. If found effective and safe, the results of the DREAM study would be a major advance in the field and a game changer in the management of chronic sciatica. Duloxetine will be the only simple, low-cost, intervention with robust and definitive evidence of effectiveness for chronic sciatica.
