# Supplementary material for: DREAM: an adaptive, randomised, placebo-controlled trial of duloxetine for reducing leg pain in people with chronic sciatica—trial protocol
Source: BMJ Open. 2024 Dec 31;14(12):e096796. doi: 10.1136/bmjopen-2024-096796 (PMC11749743; doi:10.1136/bmjopen-2024-096796)
Supplement: Supplementary file 1 [file bmjopen-2024-096796.reviewer_comments.pdf]

PEER REVIEW HISTORY

BMJ Open publishes all reviews undertaken for accepted manuscripts. Reviewers are asked to complete a checklist review form and are provided with free text boxes to elaborate on their assessment. These free text comments are reproduced below.

ARTICLE DETAILS

Title (Provisional)

DREAM - an adaptive, randomised, placebo-controlled trial of duloxetine for chronic sciatica: trial protocol.

Authors

McLachlan, Hanan; Maher, Chris; Lin, Christine; Billot, Laurent; Day, Richard O; Ivers, Rowena; Underwood, Martin; McLachlan, Andrew; Richards, Bethan; Finnerup, Nanna; Ferreira, Giovanni E

This article was not externally reviewed at BMJ Open. Protocols that have been independently assessed prior to submission to BMJ Open are usually fast-tracked to publication on the grounds that further substantial changes will not be possible. This independent assessment will usually be external, independent review for both a substantial grant award from a non-commercial or government funder and ethics approval.
